# Generation and application of drug indication inference models using typed network motif comparison analysis

**DOI:** 10.1186/1472-6947-13-S1-S2

**Published:** 2013-04-05

**Authors:** Jaejoon Choi, Kwangmin Kim, Min Song, Doheon Lee

**Affiliations:** 1 Department of Bio and Brain Engineering, KAIST, Daejeon, South Korea; 2 Department of Library and Information Science, Yonsei University, Seoul, South Korea

## Abstract

**Background:**

As the amount of publicly available biomedical data increases, discovering hidden knowledge from biomedical data (i.e., Undiscovered Public Knowledge (UPK) proposed by Swanson) became an important research topic in the biological literature mining field. Drug indication inference, or drug repositioning, is one of famous UPK tasks, which infers alternative indications for approved drugs. Many previous studies tried to find novel candidate indications of existing drugs, but these works have following limitations: 1) models are not fully automated which required manual modulations to desired tasks, 2) are not able to cover various biomedical entities, and 3) have inference limitations that those works could infer only pre-defined cases using limited patterns. To overcome these problems, we suggest a new drug indication inference model.

**Methods:**

In this paper, we adopted the Typed Network Motif Comparison Algorithm (TNMCA) to infer novel drug indications using topology of given network. Typed Network Motifs (TNM) are network motifs, which store types of data, instead of values of data. TNMCA is a powerful inference algorithm for multi-level biomedical interaction data as TNMs depend on the different types of entities and relations. We utilized a new normalized scoring function as well as network exclusion to improve the inference results. To validate our method, we applied TNMCA to a public database, Comparative Toxicogenomics Database (CTD).

**Results:**

The results show that enhanced TNMCA was able to infer meaningful indications with high performance (AUC = 0.801, 0.829) compared to the ABC model (AUC = 0.7050) and previous TNMCA model (AUC = 0.5679, 0.7469). The literature analysis also shows that TNMCA inferred meaningful results.

**Conclusions:**

We proposed and enhanced a novel drug indication inference model by incorporating topological patterns of given network. By utilizing inference models from the topological patterns, we were able to improve inference power in drug indication inferences.

## Background

**Figure 1:**
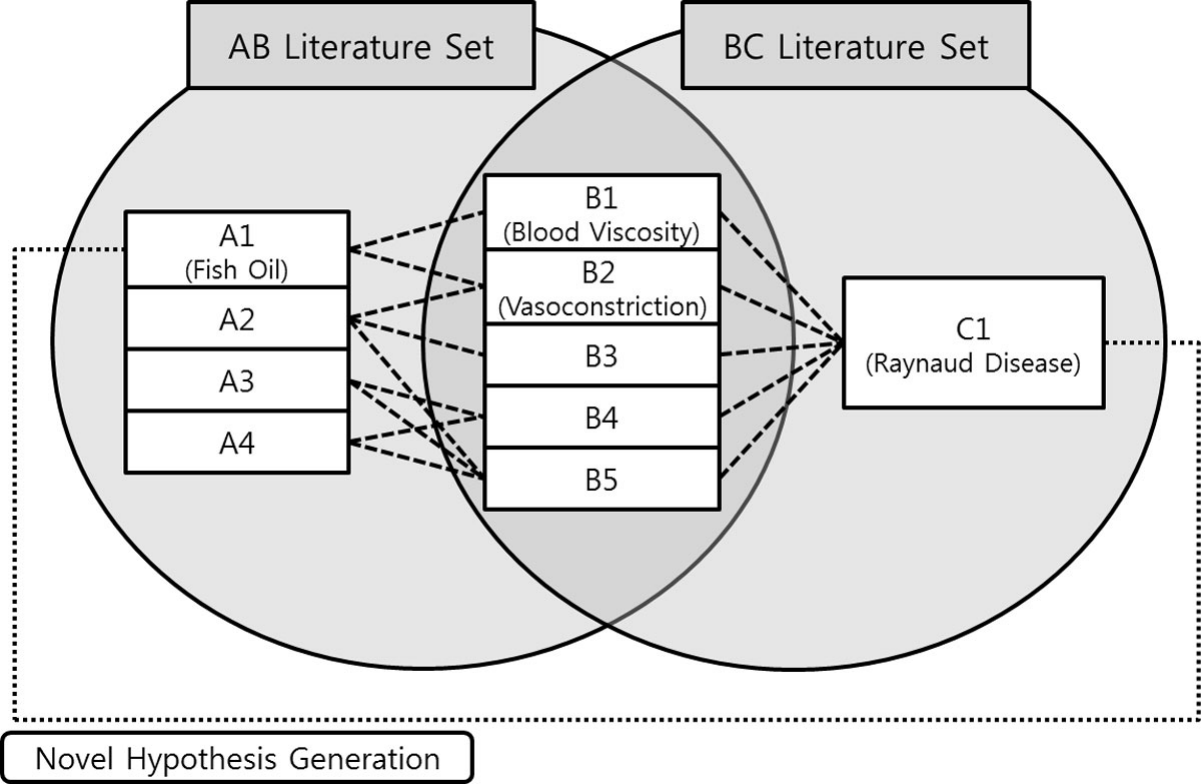
**Swanson's UPK model**. Swanson proposed the UPK model to discover the implicit relations among biological entities. The figure shows how Swanson inferred relationship between fish oil and Raynaud disease.

In 1986, Swanson proposed Undiscovered Public Knowledge (UPK) as an undiscovered knowledge which can be inferred by considering two (or more) complementary public relations [1]. UPK model is also said to be ABC model, because it implies that, even though there is no interaction between the entity A and the entity C, if there are associations between A and B, and between B and C, a new relation between A and C can be inferred. (See Figure 1.) Using the method, Swanson inferred several interactions. One of the inference results was the interaction between 'Fish oil' and 'Raynaud's Disease'. After three years, this hypothesis was proved by DiGiacomo clinically [2].

There were many attempts to improve UPK model. Hristovski, et al. [3] utilized the MeSH descriptors as features, and searched co-occurrence of the words. Pratt and Yestisgen-Yildiz [4] used Unified Medical Language System (UMLS) concepts as features, and searched only the titles as a starting concept. To reduce the number of concepts, they categorized the terms. Lee, et al. [5] utilized context terms to achieve better precision.

Drug indication inference, also known as drug repositioning, is one of famous UPK tasks. It infers alternative indications for approved drugs. By using conventional pharmacology techniques, developing a new drug requires high costs both in time and money. [6] Aided by the growth of computation power, repositioning approved drugs to new indications has decreased development costs of drugs.

Previous works of drug indication inference utilized various biological data sources to infer novel drug indications. Lamb, et al. [7] used the Connectivity Map (CMap) which ranks drug response gene expression profiles. Chiang and Butte [8] proposed the Guilt by Association (GBA) which suggests new indications by assuming that if two diseases have same indication, another drug which treats only one of them could treat the other. Gottlieb, et al. [9] proposed utilized drug-drug and disease-disease similarity measures in predicting drug indications.

These previous studies had several limitations. First, most of them are not fully automated, and required manual operations. Because of the amount of biomedical data is large, automation is critical. Second, the previous works were not performed in biomedical domains. It is necessary to consider the characteristics of the data. Since biomedical data covers from molecular level entities to phenotypic level entities, the information model should account for various types. Third, most of the methods make inference based on stereotypical inference models, which are extended from Swanson's ABC model. ABC model is based on transitive inference, and it is difficult for biomedical data inference, because of the variety of entities and relations.

To overcome these problems, we proposed Typed Network Motif Comparison Algorithm (TNMCA). [10] TNMCA is a method for drug repositioning from a large amount of multi-level biomedical interaction networks by employing typed network motif (TNM). Network motif is introduced by Milo, et al. [11] as a pattern of connectivity that occurs significantly more frequently than expected. We evolved the concept into TNM which is more adaptable to multi-type interaction networks. Whereas ordinary network motif is based on the topological connectivity, TNM considers types of nodes as well as edges.

**Figure 2:**
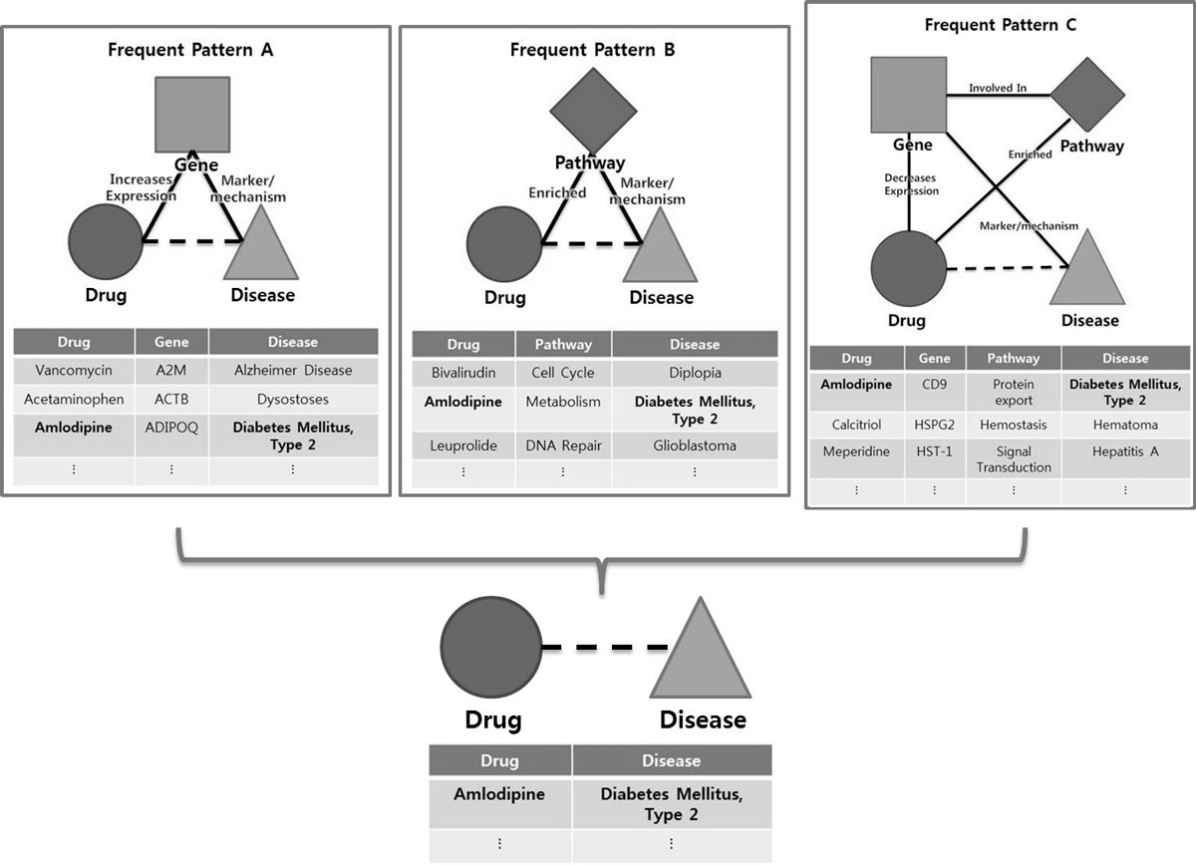
**An example of inference models**. An example with three inference models. This shows necessity of TNMCA.

Three inference models are represented in Figure 2. The leftmost model is based on the pattern composed of drug, gene, and disease. The middle model is based on the pattern composed of drug, pathway, and disease. The rightmost model is based on the pattern composed of drug, gene, pathway, and disease. If all three patterns are frequently occurred in the network, and if we integrate the inferred results from them, we can make a reliable and novel inference. These frequent patterns are TNMs in our research, and we proposed a method to extract the TNMs from the given network automatically. After extracting TNMs, we applied them to make novel inferences.

TNMCA provides several advantages over previous methods. TNMCA extracts TNM sets using the given data itself, so that it would not require any manual operation. Previous researches utilize ABC model-based transitive inferences. On the other hand, TNMCA is not limited to the transitive inferences, and is possible for the inference using generalized topological patterns. Also, TNMCA is able to infer not only interactions but also types of the interactions.

We applied two modifications to improve TNMCA. First, we enhanced scoring function of TNMCA to overcome normalization problems. Previous algorithm suffered with normalization problems that the result scores were not normalized on the size of given network and on the size of inference patterns. By limiting the score range, and modifying the summating operations, we could improve scoring function of TNMCA. Second, we limited training network to the network of associated diseases of the target disease. We filtered out unrelated diseases by disease hierarchy in Medical Subject Headings (MeSH) [12]. The diseases in the same disease hierarchy with the target disease were utilized as training set. By adopting these two improvements, we could obtain better results.

We applied enhanced TNMCA, to the publicly available database, Comparative Toxicogenomics Database (CTD) [13], to validate our method. We validated our method by comparing inference results with previous TNMCA, and ABC-based model. We used a scoring function, which depends on the frequency ratio of referenced patterns, to rank TNMCA results. The results show that enhanced TNMCA was able to infer meaningful indications with high performance compared to the ABC model and previous TNMCA model. Moreover, we confirmed that high-scored results of TNMCA results are reported in the biomedical literature. These results imply that TNMCA can make novel inferences in biomedical fields.

The rest of the paper is organized in the following order. Methods section introduces our TNMCA technique and the descriptions of the experiments. Result section reports on the experiment results. Conclusion section concludes the paper.

## Methods

### Inference model of TNMCA

Entities of biomedical data cover broad levels from the molecular level such as DNA or chemical, to the phenotypic level such as symptom or disease. Discovering the relations between different levels becomes important because they can connect various data, and generate novel information from the merged network. A few previous studies proposed specific information models to store these various types of data. Ijaz, et al. [14] defined a specific frame of entities as an information model. In this way, they could store only the targeted information. Due to its inflexibility of the model, if different types of data are included, the information model could not handle them directly, but needed manual alteration. Therefore, defining appropriate information models is one of the challenging, important issues in handling biomedical data.

We proposed and applied TNMCA to various biomedical data by using the TNM to build an inference model. (See Figure 2.) For a given graph G = (V, E), where V = {(v_1_, vt_1_), (v_2_, vt_2_), ..., (v_n_, vt_n_)} is the set of vertices with their types in G, E={(e_1_, et_1_), (e_2_, et_2_), ..., (e_m_, et_m_)| e_i_⊆{{(v_j_, vt_j_), (v_k_, vt_k_)}| (v_j_, vt_j_)⊆V, (v_k_, vt_k_)⊆V and (v_j_, vt_j_)≠(v_k_, vt_k_)}} is the set of edges with their types in G, we define the typed network motif TNM⊆{(V', E')}, where V'⊆{vt_1_, vt_2_, ..., vt_n_}, E'={(e'_1_, et_1_), (e'_2_, et_2_), ..., (e'_m_, et_m_) | e'_i_⊆{{vt_j_, vt_k_} | vt_j_⊆V', vt_k_⊆V'}}, V' is connected, count(V')>2, degree(vt_i_)>1|vt_i_⊆V'. We defined the TNM as a connected sub-graph which contains more than two node types with degree larger than one, and their connecting edges (edge types are optional). Both nodes and edges contain the types of their original values (instead of the value itself). We defined the TNM as connected, because unconnected biological concepts mean unrelated information. We set the minimum number of nodes to three because a two-node sub-graph merely represents a relation not a pattern. The minimum degree of nodes is limited to two because we cannot infer relations from one-degree nodes.

The main objective of the TNM model is to find frequent network patterns from the given network. To this end, we proposed a concept, TNM, which was evolved from the ordinary network motif. The ordinary network motif is based on topology of given network. In contrast, the TNM utilized the types of nodes and edges, so that it could be applied to multi-level biological networks.

TNM nodes and edges contain entity types instead of values. Using entity types instead of values gives novelty to the inference model. Value-based models are limited to make inferences using sub-networks with same values. In contrast, the TNM model can be applied to sub-networks with different values if the sub-networks have same type topologies.

**Figure 3:**
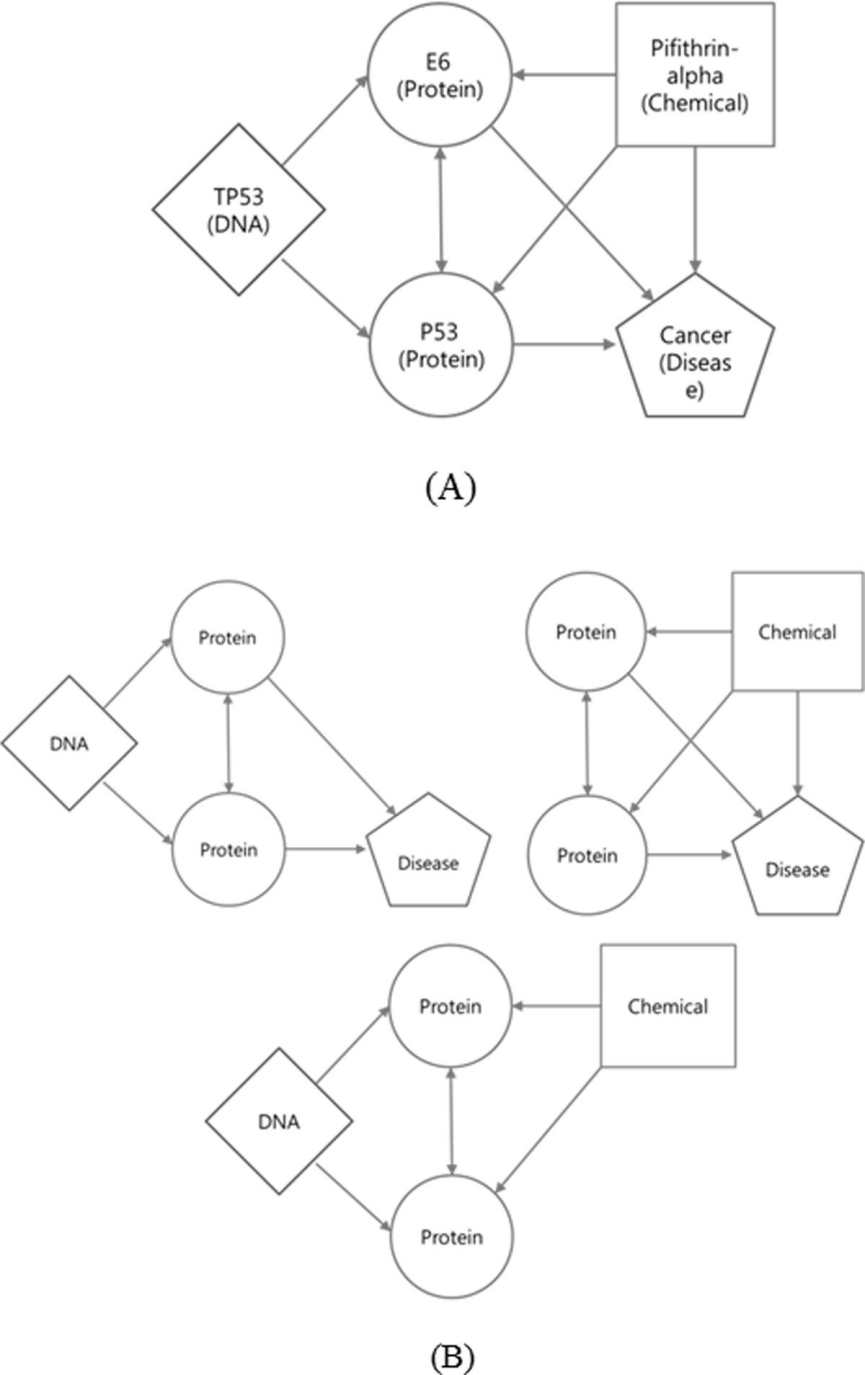
**Concept description of TNM**. (A) An example relational network (relation value not indicated) and (B) its possible four-node TNMs. TNMs use only types of nodes and types of relations (not their values).

In Figure 3(A), which represents an example relational network, nodes contain their values (such as TP53, P53, E6, Pifithrin-alpha, and Cancer), but in Figure 3(B), which shows the network's possible three-node TNMs, nodes contain their types (such as DNA, protein, chemical, and disease) only.

The TNM is a flexible information model that can be applied to various types of information. The TNM does not require any manual operation coupled with data. If the model is to be applied to different data, all it takes is to adjust the composing types of entities and relations. Using the TNM as an information model of TNMCA made the method to be applicable to various biomedical data.

### TNMCA architecture

**Figure 4:**
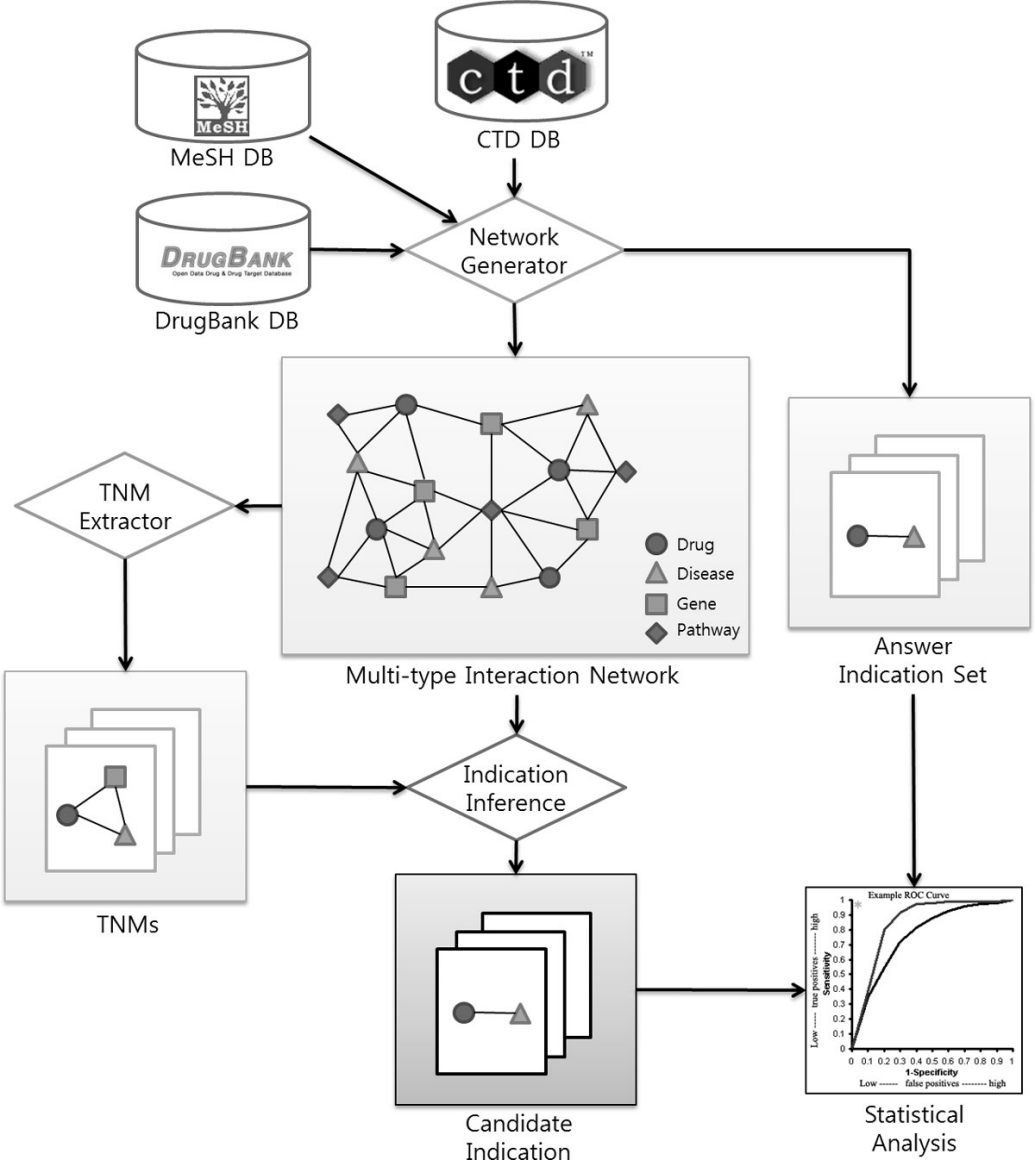
**Overall workflow of TNMCA**. TNMCA consists of four modules; Network Generator, TNM Extractor, Indication Inference and Statistical Analysis.

Figure 4 shows the overall workflow of TNMCA. TNMCA consists of four major modules; Network Generator, TNM Extractor, Indication Inference, and Statistical Analysis modules.

Network generator constructs a backbone multi-type interaction network by integrating interaction information from CTD database[13]. During construction, non-drug chemicals are excluded by referencing DrugBank database [15], and unrelated diseases of target disease are excluded by referencing MeSH disease hierarchy [12]. (See Experiment section for precise description of the database.) In validation, we set our task to find drugs for 'Type 2 Diabetes Mellitus (T2DM)'. Therefore, during generating the multi-type interaction network, we excluded chemical-disease interactions which include T2DM. We separated them as an answer indication set, and compared it with the inferred results.

TNM extractor extracts TNM from the backbone multi-type interaction network. The extractor detects every TNMs existing in the network. It checks every possible connected k-node sub-graphs, and transforms them into TNM. The TNMs and their frequencies in the network are stored as the TNM set. The frequency is used as a significance measure of each pattern. If a TNM has high frequency, then it can infer confident knowledge.

Indication inference is performed by identifying new indication candidates from the multi-type interaction network using the extracted TNM set as inference models. We search similarities between the extracted TNMs and every possible connected sub-graph of the network. If all of the parts of a sub-graph except one relation are matched with one pattern of the TNM set, we can infer the relation as new knowledge. To rank drug indication candidates, every drug indication candidates are scored. The TNM is scored by calculating proportion of the frequency of the TNM, and the inferred drug indications are scored as below:

$$\text{Score}\left(\text{TN}{\text{M}}_{\text{i}}\right)=\frac{\text{Freq}\left(\text{TN}{\text{M}}_{\text{i}}\right)}{\text{Freq}\left(\text{TN}{\text{M}}_{\text{total}}\right)}$$

$$\text{Score}\left(\text{ln}{\text{d}}_{\text{j}}\right)=1-\prod_{\text{i}}^{\text{Referenced}}\left(1-\text{Score}\left(\text{TN}{\text{M}}_{\text{i}}\right)\right)$$

where i represents an index of TNMs, and j represents an index of indications. As the score of TNMs are defined as proportion of the frequency of the TNM, the range of the score is limited from 0 to 1. For example, if a drug indication candidate is inferred from 20% frequent pattern and 50% frequent pattern, then, the score of the drug indication candidate is

$$\text{Score}=1-\left(1-0.2\right)∙\left(1-0.5\right)=0.6.$$

By using the equation, the indications always have bigger scores than the scores of every referenced TNMs. If two or more indications have same scores, the indication which has higher frequency of referenced patterns will go to higher rank. After scoring, the drug indication inference system removes unimportant drug indication candidates depending on the score of them with an appropriate score threshold.

Statistical analysis is performed by comparing inferred candidate indications with the answer indication set. By drawing receiver operating characteristic (ROC) curve based on the inference score, we could calculate area under curve (AUC) value of ROC curve for the inference results. ROC curve is created by plotting true positive rate (TPR) on false positive rate (FPR) at various thresholds of the score. The AUC value of ROC curve represents how positive results are ranked higher than negative results.

### Inference model generation/application

**Figure 5:**
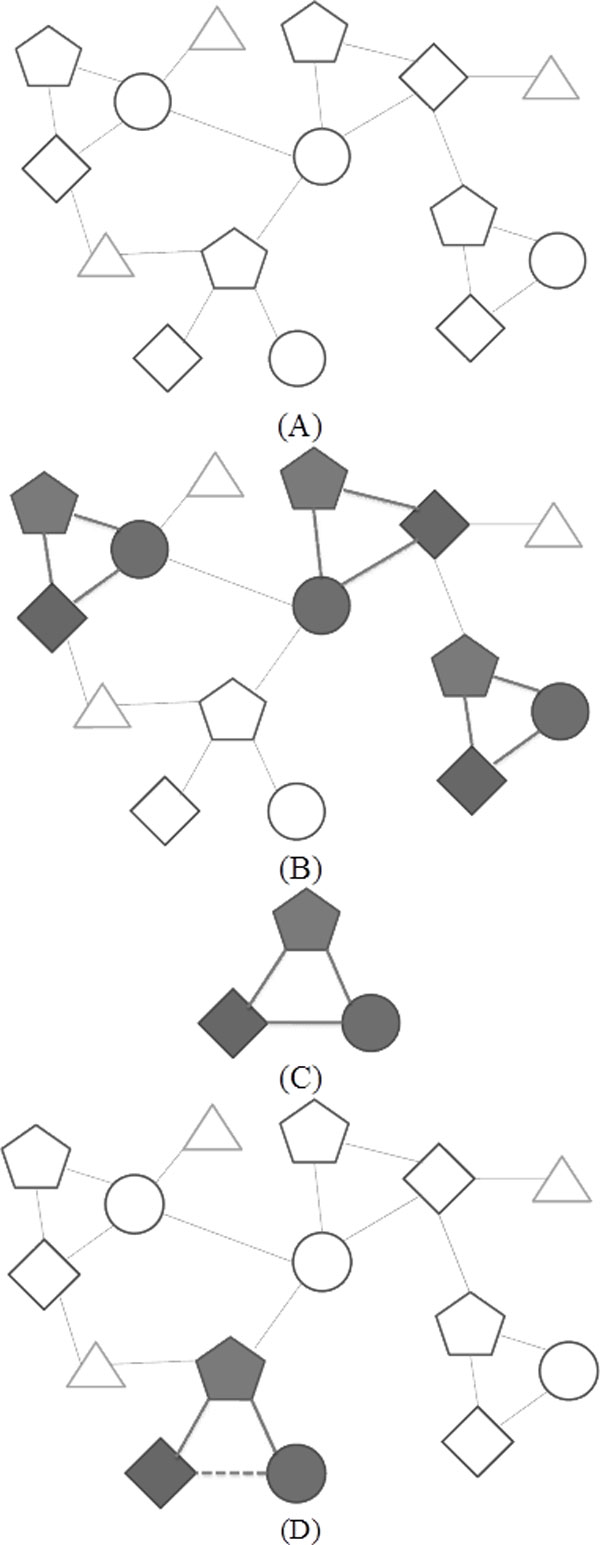
**Examples to explain inference model generation and application of TNMCA**. (A) An example multi-typed relational network (relation value not indicated). (B) A frequent TNM of the network is colored. (C) Generated inference model from the frequent TNM of the network. (D) An inference of a new relation (dotted edge).

Our target is a multi-typed interaction network. (See Figure 5(A).) A shape of each node illustrates the type of each node. An edge between two nodes means those two nodes have a specific relation. (In this example, values and types of edges are not indicated.)

A frequent TNM in the network is treated as an important one to represent a reasonable logic in the network. (See Figure 5(B), and 5(C).) We utilized the TNMs as our inference models to make inferences. Frequent patterns are different for each network. TNMCA searches and records these patterns with their frequency.

If there is a sub-network, which is similar with the frequent pattern, we infer a new candidate relation from the difference. (See Figure 5(D).) If there is a frequent pattern in a specific network, it means there is a higher probability for the network to have same patterns. Therefore, if there is a sub-network similar with the pattern, we can infer probable relations from it.

With the Swanson's model, suppose that the rectangular node is a chemical, the pentagonal node is a physical phenomenon, and the circle node is a disease. Swanson's assumption was that if a specific chemical affects a specific physical phenomenon, and the phenomenon affects a specific disease, then the chemical could affect the disease. This assumption is applied to the frequent TNMs in the network.

The Swanson's model could define appropriate logics for different networks. However, it requires filtering the concepts or relations, and it may also need to define a new transitive rule for complex networks. On the other hand, TNMCA searches frequent patterns for each network to discover appropriate patterns for each network. Since these patterns are embedded in the given data sets, unlike the Swanson's model, it does not need any optional logic definition.

### Experiments

We used Comparative Toxicogenomics Database (CTD, http://ctdbase.org/) as a database for generating a backbone multi-type interaction network[13]. CTD contains manually curated and inferred interaction data among diseases, chemicals, genes, and pathways. We used these four biological entities as the types of nodes, and their interaction types as the types of edges (if available). We excluded GO entity, because CTD provides only chemical-GO associations and no other associations with GO. Currently, CTD contains 560,956 chemical-gene associations, 9,919,586 gene-disease associations (22,446 curated and 9,897,140 inferred), 1,015,365 chemical-disease associations (175,272 curated and 840,093 inferred), 201,288 chemical-pathway associations, 43,970 disease-pathway associations, and 62,254 gene-pathway associations.

Using disease hierarchy from the Medical Subject Headings (MeSH, http://www.nlm.nih.gov/mesh/) database, we can discriminate unrelated diseases of the target disease from backbone network. MeSH is a hierarchical vocabulary containing 26,853 descriptors.

We referenced the DrugBank (DrugBank, http://www.drugbank.ca/) database to filter out non-drug chemicals from the backbone interaction network [15]. DrugBank offers 6,711 drug information with their targets. To eliminate redundant interactions which include non-drug chemicals from CTD database, we selected chemical interactions which have chemicals registered in DrugBank.

We performed our evaluation by comparing our enhanced TNMCA model with the previous TNMCA model and basic ABC model. CTD database contained 25 drugs which have T2DM as their indications. The indications were removed from input data, and both model needed to find them from rest of input data.

ABC model inferred the results by connecting drug-gene and gene-disease interactions or drug-pathway and pathway-disease interactions. If a drug is connected to the 'T2DM' through any gene or pathway, the drug becomes an inferred indication of 'T2DM'. The counts of the inferred indications were assigned as their scores.

Both TNMCA models extracted 3-node and 4-node TNMs from the backbone network.

After we get results from all models, we compared them with the answer set (25 indications of T2DM). Because ABC model cannot infer interaction types, we rated the result correct if there is the result interaction in the answer set. (Regardless of interaction types) On the other hand, for TNMCA models, the result interactions should match their types also. We calculated AUC values of the ROC curves of the results.

## Results

### TNM results

**Figure 6:**
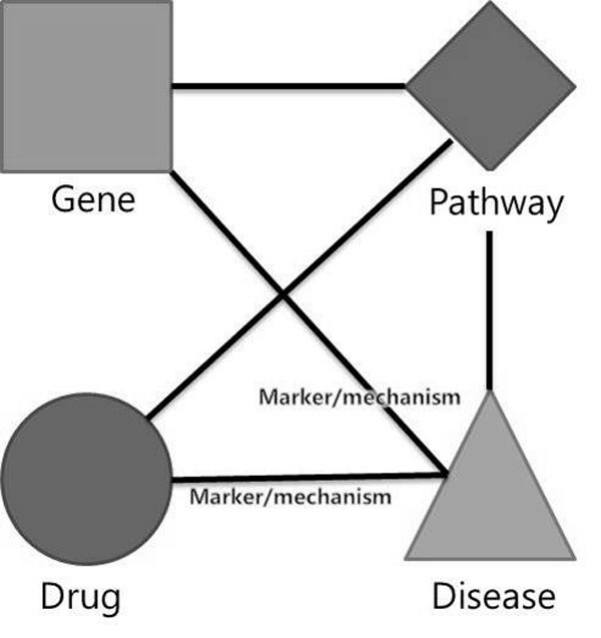
**The most frequent 4-node TNM**. The 4-node TNM which has the highest frequency.

We found unique 292 3-node TNMs, and 2,195 4-node TNMs in the network. The 4-node TNM which has the highest frequency is represented in Figure 6. As only gene-disease, drug-disease, and drug-gene interactions have interaction types, rest interaction types are not represented in Figure 6. We can find that the top-rated TNM does not have interaction between gene and drug. This indicates that many drugs for a certain disease have interaction with disease-related pathways, not disease-related genes, which supports the claim of Pujol, et al.[16] that pathway is more important than single protein in multifactorial diseases.

### Inference results

**Figure 7:**
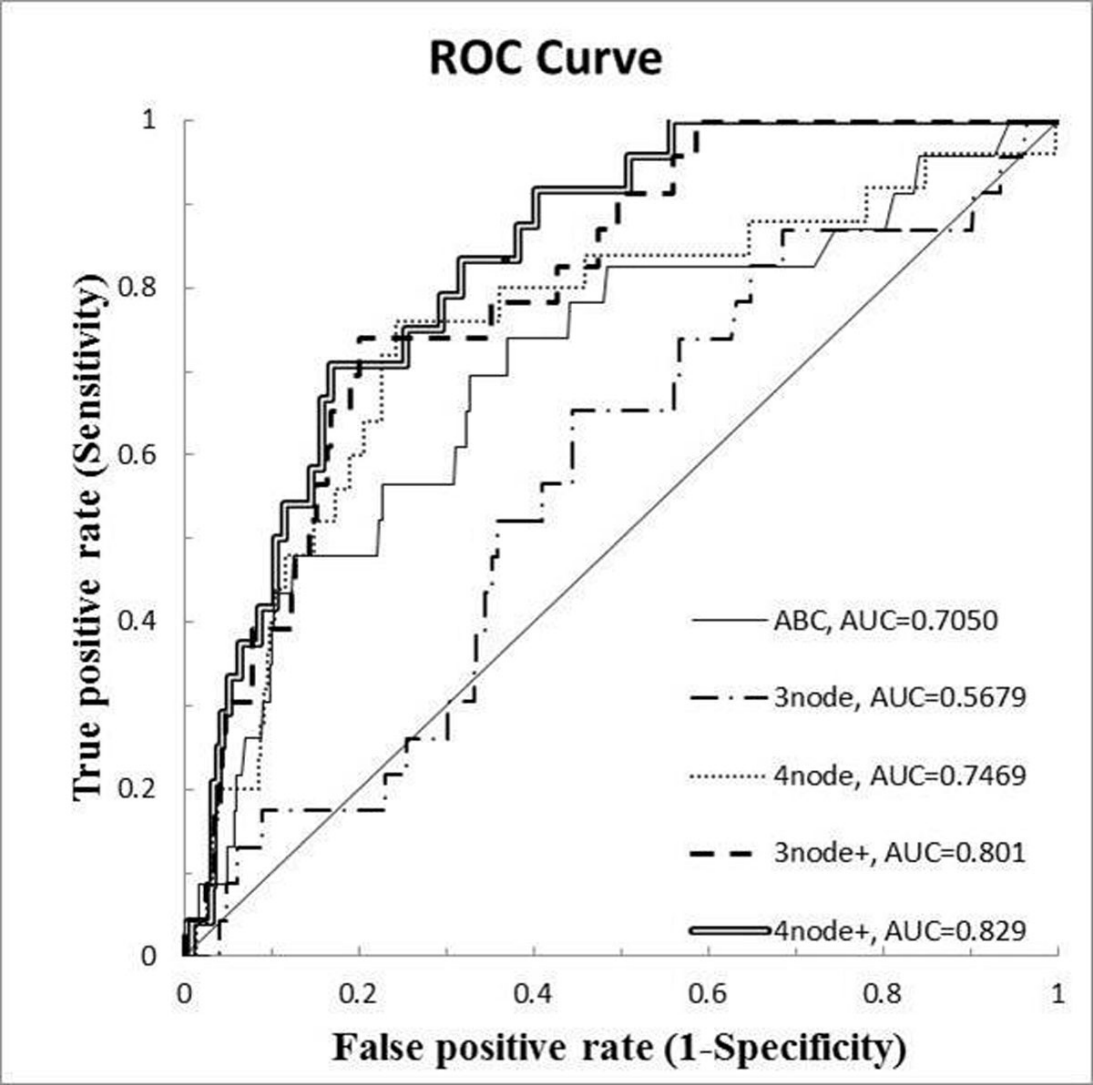
**Result of the experiments**. ROC curves of five models are plotted with their AUC value. ABC represents basic ABC model. 3node and 4node represents previous TNMCA models. 3node+ and 4node+ represents enhanced TNMCA models.

Figure 7 shows the result of our experiments. The graph shows ROC curves of ABC model results, previous 3-node TNMCA model results, previous 4-node TNMCA model results, enhanced 3-node TNMCA model results, and enhanced 4-node TNMCA model results respectively according to their AUC values. The AUC values of ABC model, previous 3-node TNMCA model, previous 4-node TNMCA model, enhanced 3-node TNMCA model, and enhanced 4-node TNMCA model are 0.7050, 0.5679, 0.7469, 0.801, and 0.829, respectively. We can find that our enhanced model improved the AUC results in both 3-node TNMCA model and 4-node TNMCA model. Previous 3-node TNMCA model had lower AUC value than ABC model, but enhanced 3-node TNMCA model resulted higher AUC value, in contrast. We should consider that TNMCA model needed to match interaction types also, whereas ABC model required the existence of the interaction only. As inference power of TNMCA increases in proportion to size k, the AUC results of them are proper. It is outstanding that enhanced TNM showed higher AUC value, even though it needed to match interaction types. This supports the inference power of TNMCA.

It would be better if we could have experiments with larger k values (larger than 4) of TNM, because many novel inferences will be performed using large k values. The AUC values also support this claim because AUC value of 4-node TNMCA results was higher than that of 3-node TNMCA results. Unfortunately, the processing time increases exponentially by k. If we set k as 5, it took several months to be performed, so we could not analyse it properly in the time of submitting this paper.

### Literature analysis

We conducted a literature review on the top 10 highest score results of enhanced 4-node TNMCA model. (See Table 1.) The table shows related literatures for the top 10 highest scored indications of 'T2DM' from enhanced 4-node TNMCA model. We searched evidences of interaction between the top 10 drugs and 'T2DM'. 'Cyclosporine' is duplicated, because there were two inferences with different interaction types. We were able to find evidences for 7 of 10 results from literature. According to the results, high-scored inferred relations from TNMCA have high reliability. The rest of results, 'Azacitidine', 'Tretinoin', and 'Daunorubicin' can be treated as newly inferred indications of 'T2DM'.

**Table 1:** Literature analysis result.

Rank	Chemical Name	CasRN	Literature Title
1	Cyclosporine	59865-13-3	The concentration of cyclosporine metabolites is significantly lower in kidney transplant recipients with diabetes mellitus. [17]
2	Heparin	9005-49-6	Diabetes Mellitus, Glycoprotein IIb/IIIa Blockade, and Heparin. [18]
3	Cholecalciferol	67-97-0	Effect of cholecalciferol supplementation on blood glucose in an experimental model of type 2 diabetes mellitus in spontaneously hypertensive rats and Wistar rats. [19]
4	Azacitidine	320-67-2	Newly inferred indication.
5	Cysteine	52-90-4	Plasma total homocysteine and cysteine in relation to glomerular filtration rate in diabetes mellitus. [20]
6	Chenodeoxycholic Acid	474-25-9	In the search for specific inhibitors of human 11beta-hydroxysteroid-dehydrogenases (11beta-HSDs): chenodeoxycholic acid selectively inhibits 11beta-HSD-I. [21]
7	Diethylstilbestrol	56-53-1	The effect of diethylstilbestrol upon alloxan diabetes in the male rat. [22]
8	Tretinoin	302-79-4	Newly inferred indication.
9	Daunorubicin	20830-81-3	Newly inferred indication.
10	Ursodeoxycholic Acid	128-13-2	Chemical Chaperones Reduce ER Stress and Restore Glucose Homeostasis in a Mouse Model of Type 2 Diabetes. [23]

Related literatures for the top 10 highest scored indications of 'T2DM' from enhanced4-node TNMCA model. Cycloporine [17], Heparin [18], Cholecalciferol [19], Cycteine [20], Chenodeoxycholic Acid [21], Diethylstilbestrol [22], and Ursodeoxycholic Acid [23] were reported to have interactions with 'T2DM'.

## Conclusions

Studies on drug repositioning have recently been rigorously carried out, and it is a difficult challenge to infer novel drug indications from a large amount of multi-level biomedical interaction networks. We proposed a novel automated inference method for various types of biomedical data. The method exports typed network motifs of data, and infers novel hypotheses by comparing the exported typed network motifs with the sub-graph of data. The method was applied to CTD database, and it achieved the outstanding performance.

The contribution of this paper is two-fold: 1) We developed a pattern finding model which can extract more generalized patterns than ABC model to solve UPK problems. Most UPK models are depending on ABC patterns or extension of ABC patterns. We propose more generalized patterns to make novel inferences. 2) We proposed an inference method which could infer interactions as well as their interaction types whereas ABC model could infer only the existence of interactions between two entities.

As a follow-up study, we plan to apply TNMCA to more complex database. CTD contained only 4 types of entities and a few types of interactions. As TNMCA is dependent on the network topology and their types, applying it to complex database will make outperforming results. Currently, a complex database is not available. Therefore we plan to integrate several biomedical databases and electric health records to construct complex multi-type interaction network for TNMCA.

## Competing interests

The authors declare that they have no competing interests.

## Authors' contributions

JC designed the method, performed experiments and drafted the manuscript. KK carried out statistical analysis for validation. MS participated in experiment design and coordination and drafted the manuscript. DL supervised the study and revised the manuscript. All authors read and approved the final manuscript.

## Declarations

The publication costs for this article were funded by the corresponding author.

This article has been published as part of *BMC Medical Informatics and Decision Making *Volume 13 Supplement 1, 2013: Proceedings of the ACM Sixth International Workshop on Data and Text Mining in Biomedical Informatics (DTMBio 2012). The full contents of the supplement are available online at http://www.biomedcentral.com/bmcmedinformdecismak/supplements/13/S1.
